# The Efficacy, Safety, and Economic Outcomes of Using Ferric Derisomaltose for the Management of Iron Deficiency in China: A Rapid Health Technology Assessment

**DOI:** 10.7759/cureus.48717

**Published:** 2023-11-13

**Authors:** Lu Yun, Zhu YuMei, Vallish BN, Qingu Tang, Chang Feng

**Affiliations:** 1 School of International Pharmaceutical Business, China Pharmaceutical University, Nanjing, CHN; 2 Department of Health Economics and Outcomes Research, Biostatistics, and Medicine, MarksMan Healthcare Communications, Hyderabad, IND

**Keywords:** cost-effectiveness, safety, efficacy, rapid health technology assessment, iron sucrose, ferric carboxymaltose, ferric derisomaltose, iron deficiency

## Abstract

Intravenous (IV) iron supplementation is the preferred treatment option for managing severe iron deficiency (ID) and ID anemia (IDA). Three of the available IV iron preparations are ferric derisomaltose (FDI), ferric carboxymaltose (FCM), and iron sucrose (IS). The objective of the present work was to review the published literature about the efficacy, safety, quality of life (QoL), and economic outcomes of using FDI, FCM, and IS for the treatment of ID. A systematic literature search was performed following Preferred Reporting Items for Systematic Reviews and Meta-Analyses (PRISMA) guidelines. Eligible studies were assessed for quality using appropriate tools, and data were extracted and analyzed for key outcomes. The evidence synthesis was based on published systematic literature reviews (SLRs), meta-analyses (MAs), indirect treatment comparisons (ITCs), and health technology assessments (HTAs); we also included economic evaluations performed from a Chinese perspective. Out of 337 initial hits, the review included 12 studies. The findings indicated that FDI, FCM, and IS had comparable efficacy in terms of hemoglobin (Hb) improvement. FDI showed a better safety profile with a lower risk of hypophosphatemia, hypersensitivity reactions, and cardiovascular adverse events (AEs) compared to IS and FCM. FDI also demonstrated better cost-effectiveness compared to IS, with potential cost savings attributed to fewer infusions and improved compliance. None of the included studies evaluated QoL after IV iron administration for ID. FDI offers a safe, efficacious, and cost-effective treatment option for ID. It exhibits comparable efficacy to FCM and IS but presents a better safety profile and economic advantage. FDI fulfills the criteria of efficacy, safety, economy, innovation, suitability, and accessibility, making it a promising choice for ID management in China.

## Introduction and background

Iron deficiency (ID), characterized by insufficient iron levels in the body, is the most common nutritional deficiency worldwide. In 2016, it was estimated that ID and its associated ID anemia (IDA) affected more than 1.2 billion people across the world [1]. The World Health Organization (WHO) acknowledged that half of the global cases of anemia are due to ID [2]. In China, based on available epidemiological data, it is believed that 70%-90% of anemia cases stem from ID [3].

Iron is an essential micronutrient: sufficient iron levels are required for numerous physiological functions, including the formation of oxygen-carrying hemoglobin (Hb) in red blood cells, myoglobin in muscle cells, and energy formation processes in all cells [1]. As a result, ID and IDA serve as independent causes of a plethora of health outcomes; additionally, as comorbidities, they can worsen the prognosis and the disease burden in conditions such as congestive heart failure (CHF), chronic kidney disease (CKD), inflammatory conditions, and cancer. ID and IDA have also been reported to increase the risks of hospitalization and death, affect maternal and fetal pregnancy outcomes, and interfere with neurocognitive development among children; preoperative anemia and ID are also recognized as contributory factors in postoperative mortality and morbidity [4-9]. Finally, ID and IDA, even in the absence of disease, are known to impair physical exercise performance and reduce quality of life (QoL). Considering the substantial impact on health, IDA has been acknowledged as one of the top five contributors to years lived with disability across the world [1,10]. Despite these serious consequences, ID remains under-recognized [11,12].

Oral iron is the recommended first-line treatment for ID primarily because of its convenience. However, only small amounts can be absorbed orally, and oral iron is associated with a high incidence of gastrointestinal (GI) side effects. Thus, oral iron might not be effective in patients with poor gastrointestinal (GI) absorption of or intolerance to oral iron, or if they require rapid repletion of iron stores [13,14]. On the other hand, IV iron bypasses the GI tract, securing efficient delivery of iron to the circulation. It is therefore the primary treatment for ID in conditions associated with oral iron intolerance, a need for rapid supplementation (e.g., perioperatively or after major blood loss), and a need to bypass the GI tract. IV iron is often the first-line treatment of IDA in patients with CKD, inflammatory bowel disease (IBD), and cancers; IV iron is also an effective supportive therapy to erythropoiesis-stimulating agents [15-18]. IV administration of iron is associated with significantly fewer GI side effects and ensures considerably faster iron replenishment compared to the oral route [19].

IV iron preparations with iron encapsulated in a carbohydrate shell or bound to a carbohydrate matrix have revolutionized iron therapy over the last decades, in comparison to the early toxic IV iron formulations, which are no longer available. The ability of IV iron preparations to delay the release of iron, thereby increasing the allowed maximum dose, depends on how tightly the iron is bound to the carbohydrate, because the stability due to this bonding limits the release of free iron during the transport of the drug via the blood to its destination in the liver macrophages [20]. Iron sucrose (IS), a second-generation IV iron preparation, consists of an iron core encapsulated in a carbohydrate shell; however, the low stability of this shell limits the dosing of IS to 100-200 mg per infusion (with a limit of three infusions per week), in order to prevent iron toxicity. Modern high-dose IV irons such as ferric derisomaltose (FDI) and ferric carboxymaltose (FCM) have a more stable structure and tighter binding of iron and consequently allow higher doses of iron to be delivered in single infusions, thereby minimizing the number of clinical contacts [21-23].

IS has been the traditionally used IV iron formulation in China [23]. The ability of IS to increase Hb in patients with IDA has been established, but optimal anemia correction and complete iron replacement with this low-dose IV iron requires multiple IS infusions; this translates to higher treatment costs both in the outpatient setting (due to the requirement of several healthcare provider visits) and in the inpatient setting (due to the requirement of prolonged hospitalization). Often, full iron correction is not achieved in clinical practice with IS due to the lack of compliance [24-26]. On the other hand, both FCM and FDI may be administered in larger doses and more rapidly than IS, allowing for a complete iron replacement within 15-30 minutes. The approved maximum dosage of FCM and FDI in China are both 20 mg/kg; there is an additional dose limitation for FCM with a maximum of 1 g per infusion and per week: this means, to cover a higher iron need using FCM, additional infusions/visits would be needed. FDI therefore has a higher allowed maximum dose that can be safely administered in a single infusion compared to FCM [20].

While FCM and FDI are both high-dose IV irons, multiple randomized controlled trials (RCTs) have reported a high incidence of hypophosphatemia, including severe and persisting hypophosphatemia, with FCM and not with FDI [27-29]. Further, observational studies demonstrate that hypophosphatemia following FCM was associated with a longer hospital stay, and treatment was often required to correct the same [30,31]. Hypophosphatemia has also been associated with several clinical manifestations, including fatigue, muscle weakness, bone pain, osteomalacia, fragility fractures, cardiovascular dysfunction, and hypoxemia due to respiratory muscle weakness [32-38]. Consequently, several authorities now require phosphate monitoring when FCM is used for ID correction in those at risk or those receiving multiple doses [39,40]. Correction of pre-existing hypophosphatemia prior to initiating FCM treatment is also required by the FDA [40].

FDI, which was previously approved in Europe (2009), Canada (2018), and the United States (2020), received approval in China in February 2021 and was included in the National Reimbursement Drug List (NRDL) in January 2023. On the other hand, FCM received approval in China in November 2022. Given this background of recent approvals of FDI and FCM and NRDL inclusion of FDI and the widespread use of IS in China, we compared these three IV iron preparations through a rapid health technology assessment (HTA). While a comprehensive HTA provides a thorough understanding of the efficacy, safety, and economic endpoints of health technologies, it can be resource-intensive and time-consuming. A rapid HTA has the advantage of being robust and evidence-driven to support informed clinical and policy-level decision-making while simultaneously not compromising on the time required for evidence synthesis and reporting [41]. Therefore, we performed this rapid HTA to quickly review the efficacy, safety, tolerability, quality of life (QoL), and economic aspects of using FDI in the management of different forms of ID, in comparison with FCM and IS. To ensure the quality of evidence synthesis in our rapid HTA was not compromised, we focused on published secondary literature reviews rather than primary trials and limited the economic evaluations to those conducted from a Chinese perspective for relevant cost-effectiveness data retrieval.

## Review

Methodology

The entire rapid HTA was performed following the 2020 Preferred Reporting Items for Systematic Reviews and Meta-Analyses (PRISMA) statement [42].

Eligibility Criteria

For our rapid HTA, we drafted the eligibility criteria based on the population, intervention, comparators, outcomes, and study design (PICOS) framework. “Population” was all patients with any form of iron deficiency, regardless of etiology, age, and sex; “intervention” was FDI monotherapy; “comparator” was therapy with FCM or IS; and “outcomes” were efficacy (improvement in hemoglobin, the number of infusions needed to bring about Hb improvement, and changes in serum ferritin, serum iron, and serum transferrin concentrations/saturations), safety and tolerability (incidence of adverse events (AEs) and serious AEs, treatment withdrawals due to AEs, special AEs such as hypophosphatemia, and mortality), patient-reported outcomes (quality of life, improvement in symptoms of iron deficiency, treatment satisfaction, patient convenience, and treatment adherence), and economic outcomes (cost associated with therapy, healthcare resource utilization, cost per quality-adjusted life year (QALY) gained, incremental cost-effectiveness ratio (ICER), and costs per utility gains). For the “study design,” we focused on secondary analyses such as systematic literature reviews (SLRs), meta-analyses (MAs), network meta-analyses (NMAs), indirect treatment comparisons (ITCs), and HTAs; for economic outcomes, we considered all forms of economic evaluations. To ensure the applicability to the Chinese context, a country restriction was applied to the economic evaluations wherein we considered only those economic evaluations that were reported from a Chinese perspective; for other types of outcomes, country restrictions were not imposed. We excluded studies that included patients without iron deficiency, pre-clinical or animal studies, and all other forms of studies not involving human participants and studies published before January 1, 2009.

Literature Search, Data Extraction, Quality Assessment, and Data Synthesis

Following the eligibility criteria described above, we developed a literature search strategy using a combination of search terms and Boolean operators, for MEDLINE/PubMed, which was modified and finalized after internal discussion. The same search strategy was adopted for Chinese National Knowledge Infrastructure (CNKI), Wanfang, and VIP databases. The detailed PubMed search strategy is available in Table 1. Using the refined search strategies, a systematic literature search was performed on May 16, 2023, in MEDLINE/PubMed (for identifying global literature) and on June 12, 2023, in the CNKI, Wanfang, and VIP databases (for identifying Chinese literature).

**Table 1:** PubMed search strategy (executed on May 16, 2023) SLRs: systematic literature reviews

Number	Query	Hits	Facet
1	“Iron Deficiencies”[Mesh] OR “iron deficien*” OR “Sideropen*” OR “Hypoferritinemi*”	30,426	Population: iron deficiency
2	“Iron Compounds”[MeSH Terms] OR “intravenous iron”[All Fields] OR “parenteral iron”[All Fields] OR “derisomaltose”[All Fields] OR “iron sucrose”[All Fields] OR “ferric carboxymaltose”[All Fields]	73,131	Intervention + all comparators
3	#1 AND #2	5,131	Population + intervention/comparators
4	systematic[sb] OR meta-analysis[pt] OR meta-analysis as topic[mh] OR meta-analysis[mh] OR meta analy*[tw] OR metanaly*[tw] OR metaanaly*[tw] OR met analy*[tw] OR integrative research[tiab] OR integrative review*[tiab] OR integrative overview*[tiab] OR research integration*[tiab] OR research overview*[tiab] OR collaborative review*[tiab] OR collaborative overview*[tiab] OR systematic review*[tiab] OR technology assessment*[tiab] OR technology overview*[tiab] OR "Technology Assessment, Biomedical"[mh] OR HTA[tiab] OR HTAs[tiab] OR comparative efficacy[tiab] OR comparative effectiveness[tiab] OR outcomes research[tiab] OR indirect comparison*[tiab] OR ((indirect treatment[tiab] OR mixed-treatment[tiab]) AND comparison*[tiab]) OR Embase*[tiab] OR Cinahl*[tiab] OR systematic overview*[tiab] OR methodological overview*[tiab] OR methodologic overview*[tiab] OR methodological review*[tiab] OR methodologic review*[tiab] OR quantitative review*[tiab] OR quantitative overview*[tiab] OR quantitative synthes*[tiab] OR pooled analy*[tiab] OR Cochrane[tiab] OR Medline[tiab] OR Pubmed[tiab] OR Medlars[tiab] OR handsearch*[tiab] OR hand search*[tiab] OR meta-regression*[tiab] OR metaregression*[tiab] OR data synthes*[tiab] OR data extraction[tiab] OR data abstraction*[tiab] OR mantel haenszel[tiab] OR peto[tiab] OR der-simonian[tiab] OR dersimonian[tiab] OR fixed effect*[tiab] OR "Cochrane Database Syst Rev"[Journal] OR "health technology assessment winchester, england"[Journal] OR "Evid Rep Technol Assess (Full Rep)"[Journal] OR "Evid Rep Technol Assess (Summ)"[Journal] OR "Int J Technol Assess Health Care"[Journal] OR "GMS Health Technol Assess"[Journal] OR "Health Technol Assess (Rockv)"[Journal] OR "Health Technol Assess Rep"[Journal] OR "network meta analysis" OR "systematic review" OR "systematic literature review" OR "meta analysis" OR "meta-analysis" OR "network meta-analysis"	635,837	SLRs and meta-analyses
5	Costs and Cost Analysis[Mesh] OR “cost benefit” OR “cost-benefit” OR “economic evaluation” OR “cost utility” OR “Cost-utility” OR “cost effectiveness” OR “cost-effectiveness” OR “cost minimization” OR “cost-minimization”	308,291	Economic analyses
6	#4 OR #5	919,970	Study designs of interest
7	#3 AND #6	370	Population + intervention/comparators + study designs
8	#7 AND (2009/1/1:3000/12/12[pdat])	315	Studies published after January 1, 2009

The pooled list of eligible articles was deduplicated and screened for eligibility over two levels. First, the titles and abstracts of all initial records were screened, and second, the full texts of all potentially eligible articles were retrieved and screened. All ineligible articles were excluded after giving reasons for exclusion, which was also based on the PICOS framework. We additionally scanned the bibliography sections of relevant articles to identify any potential articles that were missed by the search strategy. Once all the eligible records were identified and pooled, relevant data from these records were extracted from all records using a pre-drafted data extraction table. The methodological quality of the included articles was assessed using the A MeaSurement Tool to Assess systematic Reviews 2 (AMSTAR 2) scale for SLRs [43], Consolidated Health Economic Evaluation Reporting Standards (CHEERS) 2022 checklist for economic evaluations [44], International Network of Agencies for Health Technology Assessment (INAHTA) checklist for HTAs [45], and a checklist developed by Kiefer et al. [46] for ITCs and NMAs.

All data were entered in Microsoft Excel 2019 (Microsoft Corporation, Redmond, WA), and the same program was used for performing screening, data extraction, and quality assessment. Both levels of literature search, data extraction, and quality assessment of the included articles were independently performed by two researchers, and disagreements in decisions were resolved through the mediation of a third independent researcher. As the included study types were highly heterogenous, descriptive methods were used to assess outcomes, and statistical synthesis was not performed for the same reason. Since this research does not involve the collection of fresh prospective or retrospective clinical data from human subjects and is rather a systematic compilation of previously published literature, an ethics committee approval was deemed unnecessary by the authors.

Results

Study Selection and Baseline Characteristics

**Figure 1:**
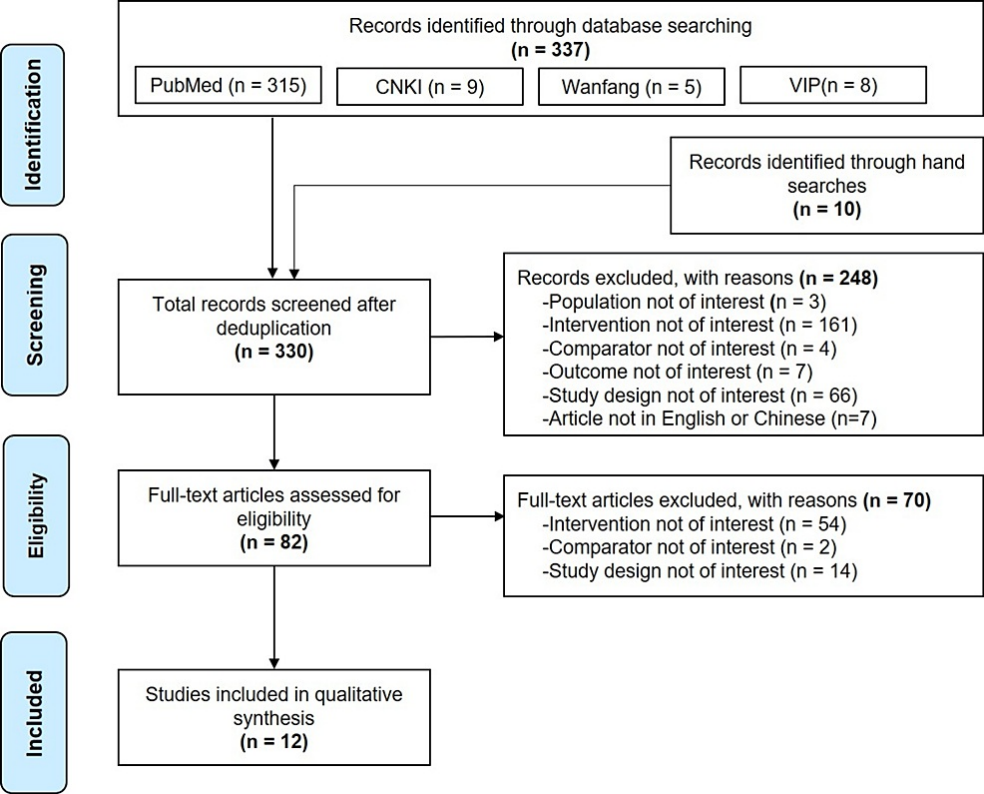
Study selection flowchart CNKI: Chinese National Knowledge Infrastructure

From an initial pool of 337 potentially eligible records from different sources, 12 studies fulfilled the eligibility criteria and were included in this HTA [47-58]. Figure 1 shows the study selection process.

Of the 12 included studies, 10 studies performed a systematic literature search; meta-analysis, network meta-analysis, and indirect treatment comparison were performed by four, four, and three studies, respectively. There was one economic evaluation and one rapid HTA among the included studies. The number of included studies within the SLRs ranged from two to 42, and the number of unique patients ranged from 594 to 11,700. Efficacy and safety were evaluated by five and nine studies, respectively; while two studies evaluated cost-effectiveness, only one study evaluated QoL. The main characteristics of the included studies are summarized in Table 2.

**Table 2:** Baseline characteristics of the included studies *Total patients may be more than the sum of patients receiving FDI, FCM, and IS because the study might have involved other forms of iron as well. AE: adverse event; CKD: chronic kidney disease; CUA: cost-utility analysis; FCM: ferric carboxymaltose; FDI: ferric derisomaltose; Hb: hemoglobin; HMB: heavy menstrual bleeding; HSR: hypersensitivity reactions; IBD: inflammatory bowel disease; ID: iron deficiency; IDA: ID anemia; IS: iron sucrose; ITC: indirect treatment comparison; IV: intravenous; MA: meta-analysis; MAIC: matching-adjusted indirect treatment comparison; MDD: maximum daily dose; MTD: mean total dose; NMA: network meta-analysis; NR: not reported; NRT: non-randomized trial; QoL: quality of life; RCT: randomized controlled trial; SAE: serious adverse event; SLR: systematic literature review; TD: total dose; WAD: weighted average dose

Study	Type of research	Country of first author	Patient profile	Dosage of IV FDI	Dosage of IV FCM	Dosage of IV IS	Type of included studies	Number of included studies	Number of patients included in the synthesis*	Parameters studied
Adler et al. (2020) [47]	SLR, NMA	Switzerland	CKD	500-1,000 mg weekly	Variable	Variable	RCTs	34	10,776 (FDI, NR; FCM, NR; IS, NR)	Efficacy: improvement in Hb level, non-response to IV iron
Aksan et al. (2017) [48]	SLR, NMA	Turkey	IBD	Mean dose: 885+238 mg (infusion)/883+ 296 mg (bolus)	Variable	Variable	RCTs, NRTs, cohort studies, case-control studies	15	1,746 (FDI, 219; FCM, 602; IS, 631)	Efficacy: improvement in Hb level; safety: incidence of AEs and SAEs
Bellos et al. (2020) [49]	SLR, NMA	Greece	IDA of multiple etiologies	1,000-2,000 mg (TD)	1,000-1,500 mg (TD)	1-2 g (TD)	RCTs	8	5,989 (FDI, 2,468; FCM, 1,252; IS, 1,155)	Safety: hypophosphatemia
Fukumoto et al. (2023) [50]	MAIC	Japan	IDA due to HMB	1,000 mg (MDD)	500 mg (MDD)	120 mg (MDD)	RCTs	2	594 (FDI, 119; FCM, 237; IS, 238)	Safety: hypophosphatemia
Hu et al. (2022) [51]	Economic evaluation	China	IDA of multiple etiologies	Variable	NR	NR	NR	NR	NR	Economic evaluation: CUA
Kennedy et al. (2023) [52]	SLR, MA	UK	IDA of multiple etiologies	NR	NR	NR	RCTs	15	10,467 (FDI, 3,474; FCM, 2,683; IS, NR)	Safety: HSR
Pollock et al. (2019) [53]	SLR, ITC	UK	IDA of multiple etiologies	1,640 mg (WAD)	1,452 mg (WAD)	Variable	RCTs	4	3,640 (FDI, NR; FCM, NR; IS, NR)	Efficacy: improvement in Hb level; safety: incidence of any AE, SAE, HP, and HSR
Pollock et al. (2020) [54]	SLR, MA, ITC	Germany	IDA of multiple etiologies	NR	NR	NR	RCTs, prospective studies	21	8,599 (FDI, 3,922; FCM, 1,775; IS, 1,503)	Safety: HSR
Pollock et al. (2022) [55]	SLR, MA, ITC	UK	IDA of multiple etiologies	1,000 mg, single dose	Two doses of 750 mg 1 week apart	800-1,000 mg	RCTs	4	6,042 (FDI, 2,008; FCM, 1,529; IS, 2,505)	Safety: mortality and cardiovascular AE
Schaefer et al. (2021) [56]	SLR, MA	Austria	ID of multiple etiologies	500-2,574 mg (MTD)	500-2,685 mg (MTD)	NR	RCTs	42	11,700 (FDI, 3,962; FCM, 7,738; IS, NR)	Safety: hypophosphatemia
Liu et al. (2022) [57]	SLR, HTA	China	IDA of multiple etiologies	NR	NR	NR	RCTs, MA, economic evaluation	9	NR	Efficacy: improvement in Hb level; safety: incidence of any AE, SAE, HP, and HSR; economic evaluation: CUA; humanistic: QoL
Huang et al. (2022) [58]	SLR, NMA	China	ID of multiple etiologies	NR	NR	NR	RCTs	15	7,524 (FDI, 2,278; FCM, 1,962; IS, 3,126)	Efficacy: improvement in Hb level and ferritin

Quality of the Included Studies

The nine studies having an SLR component were assessed through AMSTAR 2 for methodological quality. All these studies had more than two critical flaws and were judged to have a “critically low” confidence in results as per AMSTAR 2. While most studies used adequate statistical methods for meta-analysis and accounted for risk of bias (RoB) in the individual studies, none of the nine studies provided a list of excluded studies, and only two studies [47,56] investigated publication bias. The detailed results are presented in Table 3.

**Table 3:** Evaluation of methodological quality of SLRs using the AMSTAR 2 tool *Critical item AMSTAR 2: A MeaSurement Tool to Assess systematic Reviews 2 [43]; P. Yes: partial yes; PICO: population, intervention, comparator, and outcomes [43]; RoB: risk of bias; SLR: systematic literature review

Number	Items	Adler et al. (2020) [47]	Aksan et al. (2017) [48]	Bellos et al. (2020) [49]	Kennedy et al. (2023) [52]	Pollock et al. (2019) [53]	Pollock et al. (2020) [54]	Pollock et al. (2022) [55]	Schaefer et al. (2021) [56]	Huang et al. (2022) [58]
1	Did the research questions and inclusion criteria for the review include the components of PICO?	No	Yes	Yes	Yes	Yes	No	Yes	No	Yes
2*	Did the report contain an explicit statement that the review methods were established prior to the conduct of the review and did the report justify any significant deviations from the protocol?	No	No	Yes	Yes	No	No	Yes	No	No
3	Did the authors explain their selection of the study designs for inclusion in the review?	No	No	Yes	Yes	Yes	Yes	Yes	No	Yes
4*	Did the authors use a comprehensive literature search strategy?	P. Yes	No	P. Yes	P. Yes	P. Yes	No	P. Yes	No	Yes
5	Did the authors perform study selection in duplicate?	Yes	Yes	Yes	Yes	Yes	No	Yes	Yes	Yes
6	Did the authors perform data extraction in duplicate?	Yes	Yes	No	No	No	No	Yes	Yes	Yes
7*	Did the authors provide a list of excluded studies and justify the exclusions?	No	No	No	No	No	No	No	No	No
8	Did the authors describe the included studies in adequate detail?	No	P. Yes	P. Yes	No	No	No	P. Yes	P. Yes	No
9*	Did the authors use a satisfactory technique to assess RoB in individual studies included in the review?	No	P. Yes	Yes	Yes	P. Yes	No	Yes	P. Yes	Yes
10	Did the authors report on the sources of funding for the studies included in the review?	No	No	No	No	No	No	No	Yes	No
11*	If meta-analysis was performed, did the authors use appropriate statistical methods?	Yes	No	Yes	Yes	Yes	Yes	Yes	Yes	Yes
12	If meta-analysis was performed, did the authors assess the potential impact of RoB in individual studies on the results of the meta-analysis or other evidence synthesis?	No	No	Yes	No	Yes	No	Yes	Yes	No
13*	Did the authors account for RoB in individual studies during interpretation/discussion?	No	Yes	Yes	Yes	Yes	No	Yes	Yes	No
14	Did the authors account for any heterogeneity observed in the results of the review?	No	No	No	Yes	Yes	No	Yes	Yes	No
15*	If they performed a quantitative synthesis, did they investigate publication bias adequately?	Yes	No	No	No	No	No	No	Yes	No
16	Did the authors report any potential sources of conflict of interest?	Yes	Yes	Yes	Yes	Yes	Yes	Yes	Yes	No
Total number of flaws		10	9	5	6	6	13	3	5	9
Number of critical flaws		4	5	2	2	3	6	2	3	4
Confidence in results		Critically low	Critically low	Critically low	Critically low	Critically low	Critically low	Critically low	Critically low	Critically low

The assessment of the economic evaluation by Hu et al. [51] using the CHEERS 2022 checklist revealed that the authors had reported all the items in the checklist, and hence, the study has a high methodological quality. The matching-adjusted indirect treatment comparison (MAIC) by Fukumoto et al. [50] satisfied four of the nine items in the assessment checklist of Kiefer et al. [46] by establishing the research questions in advance, providing a rationale for common comparators, and describing statistical procedures and study limitations. The detailed results are available in Table 4.

**Table 4:** Quality evaluation of the matching-adjusted indirect treatment comparison using the ITC assessment scale provided by Kiefer et al. (2015) Scale adapted from Kiefer et al. [46] ITC: indirect treatment comparison

Number	Items/questions (adapted from Kiefer et al. [46])	Fukumoto et al. (2023) [50]
1	Were the questions to be addressed established in advance?	Yes
2	Is sufficient rationale given for the use of indirect comparisons?	No
3	Is sufficient rationale given for the choice of common comparators?	Yes
4	Has a complete, systematic search of the literature been performed and described in detail?	No
5	Have pre-established trial inclusion and exclusion criteria been used, and have they been clearly described?	No
6	Was a complete report of all the relevant data available?	No
7	Have the basic assumptions been examined, and have the findings of this examination been suitably handled?	No
8	Have suitable statistical procedures been used and described in detail?	Yes
9	Have limitations been sufficiently described and discussed?	Yes

Finally, the rapid HTA by Liu et al. [57] satisfied 18 of the 31 items in the INAHTA checklist and thus had moderate methodological quality. The detailed results are available in Table 5.

**Table 5:** Quality evaluation of the rapid health technology assessment using the INAHTA checklist Checklist adapted from INAHTA [45] INAHTA: International Network of Agencies for Health Technology Assessment

Number	Question	Liu et al. (2022) [57]
1	Appropriate contact details for further information?	Partial yes
2	Authors identiﬁed?	Yes
3	Statement regarding conﬂict of interest?	No
4	Statement on whether report was externally reviewed?	No
5	Findings of the assessment discussed?	Yes
6	Reference to the policy question that is addressed?	No
7	Reference to the research question(s) that is/are addressed?	Yes
8	Scope of the assessment speciﬁed?	Yes
9	Description of the assessed health technology?	Yes
10	Details on sources of information and literature search strategies provided?	
	Search strategy	Yes
	Databases	Yes
	Year range	Yes
	Language restriction	Yes
	Primary data	No
	Other kinds of information resources	Unclear
	Complete reference list of included studies	Yes
	List of excluded studies	No
	Inclusion criteria	Yes
	Exclusion criteria	Yes
11	Information on the basis for the assessment and interpretation of selected data and information	
	Method of data extraction described?	No
	Critical appraisal method (for quality assessment of the literature) described?	Yes
	Method of data synthesis described?	No
	Results of the assessment clearly presented, for example, in the form of evidence tables?	No
12	Medicolegal implications considered?	No
13	Economic analysis provided?	Yes
14	Ethical implications considered?	No
15	Social implications considered?	No
16	Other perspectives (stakeholders, patients, and consumers) considered?	No
17	Findings of the assessment discussed?	Yes
18	Conclusions from assessment clearly stated?	Yes
19	Suggestions for further action?	Yes

Clinical Efficacy

Five of the 12 studies included in this HTA evaluated efficacy endpoints [47,48,53,57,58].

Pollock et al. [53] conducted a comparative study in 2019 using an ITC to assess the efficacy of FCM and FDI in managing IDA. The study utilized IS as the common comparator. Combining effect estimates from four randomized controlled trials (three comparing FCM to IS and one comparing FDI to IS) involving 3,640 unique patients, the ITC indicated that both FDI and FCM significantly improved Hb levels. FDI demonstrated a larger increase in baseline Hb compared to FCM, with a mean difference of 0.249 g/dL (95% confidence interval (CI): 0.072-0.426). This corresponded to 8.5% more patients achieving a clinically relevant response with FDI compared to FCM, although this difference was not statistically significant (p=0.0891).

In an NMA reported by Adler et al. [47] in 2020, the efficacy of various iron supplements, including IS, FCM, and FDI, for managing anemia in patients with CKD was evaluated. The analysis divided patients into two groups: non-dialysis-dependent CKD (ND-CKD) and dialysis-dependent CKD (DD-CKD). The NMA synthesized data from 34 RCTs with 10,097 unique patients. Among ND-CKD patients, FCM (administered at 750-1,500 mg/month) was found to be more effective than FDI (administered at 1,000 mg/month), with an odds ratio (OR) of 0.44 and a 95% credible interval (CrI) of 0.20-0.99. However, there was no significant difference in efficacy between IS and FDI at any dosage range in the same subgroup: the OR (95% CrI) for IS 100-300 mg/month versus FDI was 0.60 (0.27-1.40), and for IS >300 mg/month versus FDI, it was 0.57 (0.26-1.27). However, among DD-CKD patients, no significant differences in efficacy were observed among FDI, FCM, and IS.

The NMA reported by Aksan et al. [48] in 2017 compared the efficacy and tolerability of various IV iron formulations used to treat IDA in patients with IBD by pooling results from five RCTs with 1,143 unique patients. While the analysis revealed that all three IV iron formulations had better hemoglobin response than oral iron, there was no statistically significant difference between the FDI, FCM, and IS with respect to the percentage of IBD patients showing normalization of Hb or increase in Hb of ≥2 g/dL. The OR (95% CrI) were 1.5 (0.72-2.9) for FCM versus FDI, 0.70 (0.48-1.0) for FCM versus IS, and 0.69 (0.34-1.4) for FDI versus FCM. However, with these results, the authors provided a ranking of the treatments according to their estimated magnitude in the Markov chain Monte Carlo run and concluded that FCM was the most effective treatment, followed by IS and FDI.

In 2022, Huang et al. [58] conducted an NMA comparing the efficacy of four IV iron agents (FDI, IS, FCM, and low-molecular-weight iron dextran) for the treatment of anemia. Their study included 15 papers, encompassing 16 relevant RCTs involving 7,524 unique patients. This study primarily focused on improvements in hemoglobin and ferritin levels as key indicators. The analysis suggested that the surface under the cumulative ranking curve (SUCRA) probability for hemoglobin improvement order was FCM>FDI>IS, but the difference was statistically not significant. Next, the SUCRA probability ranking for ferritin level improvement was FCM>IS>FDI, and the difference was statistically significant between FCM versus FDI and IS versus FDI.

A rapid HTA reported by Liu et al. [57] in 2022 evaluated the effectiveness of FDI compared to FCM and IS. The assessment included one HTA report, three systematic evaluations/meta-analyses, and five economics studies, all of which underwent descriptive analysis. This rapid HTA concluded that patients using FDI experienced a greater increase in Hb levels compared to those using FCM. However, the difference in the proportion of responding patients between the two treatments was not statistically significant. Moreover, FDI was found to be non-inferior to IS in terms of increasing and maintaining Hb levels, and there was no statistically significant difference in quality of life between the two groups.

Safety

Of the 12 included studies, safety outcomes were evaluated by eight studies. Specifically, hypophosphatemia and hypersensitivity reactions were evaluated by three and two studies, respectively; one study focused on cardiovascular safety, and the remaining studies elaborated on all adverse effects. The NMA published in 2019 by Pollock et al. [53] could not evaluate safety because of inadequate and inconsistent reporting of adverse events in the included trials.

The NMA reported by Bellos et al. [49] in 2020 evaluated the risk of hypophosphatemia (serum phosphate < 2 mg/dL) following the administration of different formulations of IV iron by pooling results from eight RCTs involving 5,989 unique patients with IDA of multiple etiologies. In this NMA, FCM was associated with a significantly higher incidence of hypophosphatemia than FDI and IS: the risk ratio (RR) (95% CrI) of comparisons was 7.90 (2.10-28.0) for FCM versus FDI and 9.40 (2.30-33.0) for FCM versus IS. However, the risk of hypophosphatemia was found to be similar between FDI and IS: RR (95% CrI) was 1.2 (0.19-6.20) for FDI versus IS. Further, FCM was associated with a higher incidence of severe hypophosphatemia (serum phosphate < 1.3 mg/dL) compared to FDI (10%-11.8% with FCM versus 0% with FDI). FCM was also associated with a higher proportion of patients with persistent hypophosphatemia at five weeks compared to FDI (41.9% with FCM versus 0.8% with FDI). The authors ranked FCM as the worst treatment presenting the highest SUCRA (99.1%) among the three IV irons.

In 2021, Schaefer et al. [56] reported a meta-analysis including 42 RCTs with 11,700 patients, evaluating hypophosphatemia occurring after treatment with FDI and FCM. The meta-analysis revealed that FCM was associated with a significantly higher incidence of hypophosphatemia than FDI (47% versus 4%; p<0.001) and a significantly greater mean reduction in serum phosphate (0.40 versus 0.06 mmol/L). Pooled hypophosphatemia rates were higher for FCM versus FDI in patients with CKD (27% versus 2%) as well as in non-CKD patients (51% versus 5%; p<0.001). Hypophosphatemia noted with FCM did not resolve for at least three months in up to 45% of patients who received FCM.

Fukumoto et al. [50] reported an anchored MAIC in 2023 to indirectly compare the incidence of hypophosphatemia with FDI versus FCM in Japanese patients having IDA associated with heavy menstrual bleeding. For this MAIC, patient-level data was considered from two head-to-head RCTs, one comparing FDI with IS [59] and the other comparing FCM with IS [60], with IS as the common comparator. The OR (95% CI) of hypophosphatemia incidence was 0.02 (0.01-0.05) for FDI versus IS (based on adjusted analysis) and 1.17 (0.62-2.22) for FCM versus IS (based on modeled analysis). Based on these two results, the anchored MAIC estimated that the OR (95% CI) of hypophosphatemia incidence was 52.5 (27.7-99.4) for FCM versus FDI, indicating that the odds of experiencing hypophosphatemia were 52.5 times higher with FCM than with FDI.

In 2020, Pollock et al. [54] reported the comparative incidence of serious or severe hypersensitivity reactions after treatment with FDI, FCM, and IS. This study included 21 RCTs with a total of 8,599 unique patients, and the outcomes were separately compared using Bayesian, frequentist naïve pooling, and adjusted approaches (via random-effects meta-analysis for FDI versus IS and ITC for FDI versus FCM). Based on all three approaches, FDI was found to have lesser odds of experiencing hypersensitivity reactions compared to FCM. Specifically, the Bayesian approach yielded an OR (95% highest posterior density interval (HDI)) of 0.41 (0.20-0.64) for FDI versus FCM and of 0.51 (0.26-0.79) for FDI versus IS, indicating that the odds of experiencing any hypersensitivity reaction was 59% lower with FDI versus FCM and 49% lower with FDI versus IS. The frequentist naïve pooling approach yielded an OR (95% CI) of 0.39 (0.23-0.68) for FDI versus FCM and of 0.49 (0.29-0.84) for FDI versus IS, indicating that the odds of experiencing any hypersensitivity reactions were 61% lower with FDI relative to FCM and 51% lower with FDI relative to IS. The adjusted approach yielded an OR (95% CI) of 0.45 (0.16-1.25) for FDI versus FCM and of 0.56 (0.23-1.37) for FDI versus IS, indicating that FDI had 55% and 44% lower odds of hypersensitivity reactions compared with FCM and IS, respectively. Based on this analysis, the authors concluded that FDI had a lower risk of serious or severe hypersensitivity reaction compared with FCM and IS.

Next, the meta-analysis reported in 2023 by Kennedy et al. [52] used both Bayesian and naïve pooling approaches to evaluate the incidence of hypersensitivity reactions after the administration of FDI and FCM in patients with IDA of multiple etiologies from 15 RCTs with a total of 10,467 unique patients. The incidence of any serious or severe hypersensitivity reactions was found to be lower with FDI (5/3,474; 0.14%) than with FCM (29/2,683; 1.08%). FDI was associated with lower odds of experiencing hypersensitivity reactions relative to FCM via both Bayesian analysis of proportions (mean OR: 0.16; 95% CI: 0.05-0.33) as well as the naïve pooling approach (OR: 0.13; 95% CI: 0.05-0.34).

In 2022, Pollock et al. [55] reported the results of a meta-analysis and ITC that estimated the relative incidence of blindly adjudicated cardiovascular endpoint (death due to any cause, non-fatal myocardial infarction, non-fatal stroke, unstable angina requiring hospitalization, congestive heart failure, arrhythmia, and protocol-defined hypertensive and hypotensive events) in IDA patients treated with FDI, IS, and FCM. Four large-scale RCTs were included in the analysis, and the safety analysis population was 6,042 patients. The random effects meta-analysis yielded an OR (95% CI) of 0.59 (0.39-0.90) for FDI versus IS, indicating that FDI significantly reduced the odds of the composite cardiovascular endpoint by 41% relative to IS. The indirect OR for FDI versus FCM was 0.53 (0.33-0.85), indicating that FDI significantly lowered the odds of the composite cardiovascular endpoint by 47% relative to FCM.

The SLR/NMA reported by Aksan et al. [48] in 2017 that focused on the efficacy and tolerability of different IV iron formulations administered for patients with IDA due to IBD and the safety sets for FDI, FCM, and IS included 223 patients (one study), 543 patients (five studies), and 471 patients (eight studies), respectively. The adverse event rates were found to be similar among the three IV iron preparations: the number and proportion of patients experiencing adverse events were 38 (17%), 65 (12%), and 72 (15.3%), respectively, with FDI, FCM, and IS. Notably, this study did not elaborate much on hypophosphatemia, hypersensitivity reactions, or cardiovascular adverse reactions specifically.

The rapid HTA reported by Liu et al. [57] in 2022 remarked that the incidence of AEs with FCM, IS, and FDI were 12%, 15.3%, and 17%, respectively, by synthesizing data from the paper by Aksan et al. (2017) [48] that was summarized previously. The authors reported that FDI had a lower incidence of hypophosphatemia compared to FCM and IS.

Quality of Life

In 2022, Liu et al. [57] in their rapid HTA reported that there was no difference in QoL between FDI and IS, based on a Canadian Agency for Drugs and Technologies in Health (CADTH) HTA report, which incorporated data from a total of four RCTs [61]. Two of these studies employed the Chronic Disease Treatment Fatigue Scale to assess fatigue, revealing no statistically significant difference in fatigue-related QoL outcomes between the FDI and IS cohorts. Similarly, the remaining two studies evaluated patients’ overall QoL using the Brief Health Status Questionnaire Scale and the Linear Analogue Self-Assessment Scale. These assessments collectively indicated a lack of divergence in QoL outcomes between patients receiving FDI and those receiving IS. None of the other studies included in our rapid HTA had reported the impact of the IV iron preparations on QoL.

Cost-Effectiveness

The cost-utility analysis (CUA) reported by Hu et al. [51] in 2022 compared the costs and benefits of FDI and IS for the treatment of IDA in China using a patient-level, discrete-time, illness-death model that estimated the hematologic response (change in Hb levels) and the incidence of cardiovascular events and hypersensitivity reactions with FDI and IS over a five-year time horizon. Input parameters and health state utilities were obtained from published RCTs, and the required number of infusions for FDI and IS was calculated using baseline bodyweight and Hb values and by referring to the respective label inserts. The costs for the analysis were obtained from published literature, fee schedules, and a physician survey and were expressed in 2021 Renminbi (RMB) and US dollars (USD). Cost-utility outcomes were modeled from a healthcare system perspective and from a societal perspective, and a monthly cycle length was adapted with a 5% discount rate for both costs and benefits.

It was observed that from a healthcare system perspective, FDI was associated with an incremental cost-utility ratio (ICUR) of RMB 24,901 (USD 5,949) per QALY gained relative to IS. Probabilistic sensitivity analysis suggested that at a willingness-to-pay threshold of RMB 72,477 (USD 17,307), which was also equal to the 2020 Chinese GDP per capita, there was a 100% likelihood that FDI would be cost-effective compared to IS. Further, from a societal perspective, FDI was associated with lower total costs than IS, indicating greater cost-effectiveness with FDI than with IS in China. Basically, while FDI had higher drug acquisition costs than IS, the cost-effectiveness of FDI was better compared to IS, primarily because of the higher iron delivered per treatment resulting in fewer IV administrations, lower administration-related costs, and lower infusion-related QoL reduction. FDI was also associated with fewer ADRs including hypersensitivity reactions. With these observations, the authors concluded that compared to IS, FDI was estimated to reduce costs associated with IV iron administration and improve infusion-related QoL outcomes, regardless of the perspective of analysis, and that by reducing societal costs associated with IDA, FDI provides good value for money in China [51].

The rapid HTA by Liu et al. [57] in 2022 reported the cost-effectiveness of FDI, IS, and FCM by reviewing a CADTH HTA report, one Chinese pharmacoeconomic evaluation by Hu et al. [51] that has already been summarized previously, and four other pharmacoeconomic evaluations conducted outside China, which are beyond the scope of the current review.

Discussion

The main findings of this rapid HTA are that while there is little difference in efficacy (in terms of improvement or normalization of Hb) between the three IV iron formulations, FDI is associated with a lower risk of hypophosphatemia, serious or severe hypersensitivity reactions, and cardiovascular adverse effects compared to FCM and IS. Further, from a Chinese health system and societal perspective, FDI appears to be more cost-effective and offers better value for money than IS in the management of IDA.

The quality of the included studies in our rapid HTA was assessed by three different scales, based on the study design. All eight studies that were assessed using the AMSTAR 2 tool received a “critically low confidence in results” grading. However, it is important to note that the AMSTAR 2 scale employs stringent criteria for the quality assessment of SLRs. This aspect of AMSTAR 2 has been previously criticized since it was found to assign low grades to highly cited SLRs published in high-impact journals [62]. Furthermore, the AMSTAR 2 scale does not differentiate grades based on the number of critical flaws noted in the assessed studies. For example, in our analysis, three studies with two critical flaws [49,52,55] and one study with six critical flaws [54] were all graded as “critically low.” Therefore, caution must be exercised when interpreting the results of the quality assessment using the AMSTAR 2 scale.

Among the five studies that assessed efficacy, the study by Adler et al. (2020) [47] primarily included trials comparing IV iron formulations to oral treatments and was thus not adequately powered to detect differences between IV iron formulations. Next, the studies by Pollock et al. (2019) [53], Huang et al. (2022) [58], and Liu et al. (2022) [57] indicated an absence of statistically significant difference in the proportion of patients responding to IV iron therapy with FDI or FCM, although a larger increase from baseline hemoglobin was observed with FDI compared to FCM. A recently reported RCT, the PHOSPHARE-IBD study, had similar efficacy findings in that when same doses of FDI and FCM were administered in a randomized trial to patients with IBD and IDA, the Hb increase was similar throughout the 70-day trial (Hb increase on day 70 with FDI: 24.9 g/L, 95% CI: 21.1-28.8; Hb increase with FCM: 25.2 g/L, 95% CI: 21.3-29.1) [28]. This reaffirms our finding that both FDI and FCM have similar clinical efficacy in terms of Hb response.

The Bayesian NMA reported by Aksan et al. (2017) [48] also suggested that the efficacy between the three IV iron compounds was statistically similar. Despite these findings, the authors concluded that FCM was the most effective IV iron formulation. The authors also discussed the better tolerability of FCM compared to FDI based on the proportion of patients experiencing adverse events but failed to elaborate on the relative incidence of hypophosphatemia, hypersensitivity reactions, and cardiovascular adverse effects between FCM and FDI, which were reported in SLRs published subsequently [49,50,52,54-56]. There were additional limitations to the paper by Aksan et al. (2017) [48], such as dissimilarities in the included RCTs, particularly regarding patient eligibility criteria, the nature of the response evaluated, methods to calculate iron need, and the definition of the primary endpoints. Hence, the efficacy and safety benefits associated with FCM compared to FDI, as reported in Aksan et al.’s paper, must be viewed with caution. With this background, it appears prudent to consider all three IV iron preparations that were evaluated in the present study to have similar efficacy in terms of hemoglobin response.

The apparently biased interpretation of the efficacy difference between FCM and FDI in the paper by Aksan et al. (2017) [48] formed the basis for three cost-effectiveness analyses of FCM versus FDI and IS, all three of which had Aksan as a co-author [63-65]. The first study, which concluded that FCM was more cost-effective than FDI and IS in Switzerland, was criticized for weak methodology, using variable formulae for calculating iron need, relying on the paper by Aksan et al. (2017) [48] to project higher efficacy with FCM, failing to consider cost savings with FDI due to fewer infusions required, and an overall bias in favor of FCM [66]. These limitations should also be considered in mind while interpreting the conclusions of the other two cost-effectiveness analyses.

In addition to these studies, there have been other economic analyses of different designs that have considered perspectives outside China, such as a resource impact model for preoperative iron deficiency anemia in Ireland reported in 2020 [67], two budget impact models reported in 2018 and 2021 comparing FDI and FCM in different European countries [68,69], and patient-level cost-effectiveness analysis of FDI and FCM in the United Kingdom reported in 2020 [70]; all these studies suggest that FDI is more cost-effective than FCM due to the higher maximum dose per infusion. Furthermore, two cost-minimization analyses published in 2011 also indicate cost savings with FDI compared to IS and FCM [71,72]. However, we did not elaborate on these studies in our current analysis, since we focused on economic evaluations based in China.

The decision to focus on economic evaluations reported from a Chinese perspective only was because of the challenges associated with comparing the results of health economic evaluations across geographies, which emerge as a result of differences in costs, clinical practice, prevalence, perspectives, and reimbursement considerations. Thus, this decision enhanced the relevance of the economic evaluation in the Chinese context. Our analysis showed that FDI has the potential to be cost-saving compared to IS in the Chinese healthcare and societal setting, based on the CUA by Hu et al. [51], which was reported prior to the NRDL approval of FDI in January 2023. This is notable because the NRDL approval of a drug is usually associated with a subsequent reduction of the market price of the drug in lieu of improved patient access to the treatment. This reduction in the price of FDI is bound to further improve its documented cost-effectiveness in the Chinese context. Further supporting the cost-effectiveness of FDI was a 2020 CADTH report that also concluded that FDI was associated with cost savings of USD 570 and 0.0026 additional QALYs compared to IS and observed that FDI dominated IS [73]. The higher cost savings observed with FDI relative to IS can be attributed to FDI’s ability to be administered at higher doses, requiring fewer infusions and resulting in faster correction of iron deficits. This leads to reduced healthcare resource utilization, fewer work days lost due to illness, and fewer adverse events with FDI [24,25,51,54,72]. By these results, we believe that FDI will also be cost-effective from the patient perspective as well compared to IS since FDI is associated with fewer hospital visits or days of hospitalization, fewer infusions and thus lower costs and QoL losses associated with infusion sessions, and fewer days of missed work due to faster ID correction. As such, a formal comparison of cost-effectiveness is yet to be performed between FDI and FCM in a Chinese setting. However, based on the available efficacy and safety results between these two compounds and given similar efficacy and assuming parity price, FDI will likely emerge as a more cost-effective alternative due to its higher permitted maximum dose (resulting in fewer clinical contact and more rapid anemia correction) and better safety profile (resulting in a non-requirement of hypophosphatemia monitoring and management).

As demonstrated in our analysis of three different systematic reviews, FCM was found to have higher rates of hypophosphatemia in patients with normal renal function and in patients with CKD [49,50,56]. While the exact mechanism is not fully understood, studies have shown that FCM increases circulating levels of biologically active fibroblast growth factor-23 (iFGF23), which triggers a cascade of biochemical changes [27,28,56,74-76]. Studies demonstrate that iFGF23-mediated hypophosphatemia is a specific effect of certain IV iron formulations such as FCM rather than a class effect of all IV irons [27,28,30,56,74]. It is postulated that the unique carbohydrate moiety in FCM increases iFGF23 concentration by inhibiting its degradation [74,77]. Subsequently, this results in severe reductions of 1,25-dihydroxyvitamin D, decreased intestinal phosphate absorption, acute hypocalcemia, and secondary hyperparathyroidism, leading to hyperphosphaturic hypophosphatemia [74,78]. Notably, iron polymaltose and saccharated ferric oxide, two formulations not currently available in China, have also demonstrated similar effects on iFGF23 [59,79]. In contrast, FDI has had no marked association with severe or persistent hypophosphatemia, potentially due to its negligible impact on iFGF23 [27-29,37,80,81]. The occurrence of hypophosphatemia with FCM has implications not only on the safety profile but also on QoL and costs. As evidenced by the PHOSPHARE-IBD study, patient-reported fatigue scores improved significantly faster and greater with FDI than with FCM, despite similar improvements in Hb. In addition, improvements in fatigue were inversely associated with greater magnitude of phosphate decreases, suggesting that the beneficial effect of IV iron on fatigue may be undermined by drops in phosphate [28]. The occurrence of hypophosphatemia with FCM is also expected to have implications on the overall cost of iron therapy due to the additional need for monitoring and treatment of hypophosphatemia [39,40], as well as the potential to increase hospital length of stay and risk of hypophosphatemic osteomalacia-related stress fractures [30,31,36,82].

In our analysis, we also observed an increase in the incidence of cardiovascular adverse events with FCM, which may, at least in part, be explained by the higher incidence of iFGF23-mediated hypophosphatemia with FCM. Hypophosphatemia-induced depletion of adenosine triphosphate (ATP) and 2,3 diphosphoglycerate (2,3-DPG) may lead to cardiomyopathy and arrhythmia [83], while elevated iFGF23 has also demonstrated direct adverse effects on myocardial structure [84,85]. However, it is worth noting that cardiovascular adverse events may also be secondary to oxidative stress and cell damage due to reactive oxygen species (ROS) generated from IV iron [55]. Further research is warranted to identify the factors contributing to the higher incidence of cardiovascular events with FCM. Lastly, while serious or severe hypersensitivity reactions are less of a concern with modern IV iron preparations compared to the older parenteral preparations, our analysis suggests that FDI has significantly lower event rates compared to FCM and IS, further demonstrating its favorable safety profile.

The superior safety profile of FDI can be attributed to an innovation in its chemistry and structure. Unlike most other IV irons, including FCM, which use branched polymers to form a carbohydrate shell, FDI consists of derisomaltose, a carbohydrate with a short linear structure of unbranched hydrogenated isomalto-oligosaccharides, with an average molecular weight of 1 kDa [20,86]. This results in a tighter binding of elemental iron within a non-ionic carbohydrate matrix, leading to higher stability and slower degradation of the iron-carbohydrate complex. As a result, FDI has lower levels of labile iron (less than 1% of the administered dose) [20,24]. Additionally, the use of carbohydrate moieties with reduced immunogenic activity may explain the lower risk of hypersensitivity reactions with FDI [24]. On the other hand, IV iron preparations such as IS that release iron faster from the iron-carbohydrate complex into the blood may result in excessive labile iron, potentially leading to toxicity. Consequently, lower doses of IS are required, necessitating multiple infusions to achieve the total dose [13].

In addition to demonstrating considerable efficacy, safety, economy, and innovation, FDI also meets the “suitability” dimension recommended by the Chinese National Health Commission’s Guidelines for the Management of Comprehensive Clinical Evaluation of Drugs, since it fulfills the criteria for “appropriateness of indications,” “optimal interval between drug doses,” and “compliance with medication” guidelines. Moreover, to alleviate the disease burden on patients, China has actively pursued a national drug negotiation policy, wherein the national health insurance authorities engage in negotiations with pharmaceutical companies to establish more reasonable and affordable prices for medications. FDI has been included in China’s 2022 National Health Insurance Drug catalog. Additionally, China has implemented a “dual-channel policy” to enhance the availability of the drugs that have undergone negotiation, and many provinces have already included FDI in the list of “dual-channel” managed drugs. Given these policies, FDI also appears to meet the “accessibility” dimension.

While our rapid HTA has provided valuable insights, it is not without limitations. Some of these include the lack of prospective registration of the review protocol, the exclusion of relevant databases such as Embase or Cochrane Library, and the inclusion of only articles reported in English and Chinese languages. Furthermore, we were unable to evaluate the impact of the included interventions on humanistic outcomes such as QoL or symptom relief, as none of the included studies assessed patient-reported outcomes in detail. However, based on beneficial aspects such as lower incidence of hypersensitivity reaction and hypophosphatemia and better treatment compliance due to fewer infusion sessions, it is anticipated that FDI will be associated with better QoL outcomes compared to FCM and IS.

## Conclusions

Due to its unique chemistry, structure, and innovative molecular characteristics, FDI appears to be a safe, effective, and cost-effective treatment option for patients with ID in China. While published SLRs do not indicate significant differences in efficacy among IV iron formulations, FDI demonstrates a better safety profile in terms of a lower incidence of hypophosphatemia, serious and severe hypersensitivity reactions, and cardiovascular adverse events. Compared to IS, FDI has advantages due to larger dosing per infusion, which translates to fewer infusions, cost savings, and better compliance. Compared to FCM, FDI has equal efficacy but higher maximum dosing as well as better safety in terms of lower incidence of hypophosphatemia and related better cardiovascular safety, resulting in both QoL and cost advantages. FDI was also more cost-effective than IS, which may be attributed to its innovative characteristics and safety profile that enable the correction of iron deficits with fewer infusions, thus reducing costs and improving compliance. According to these observations, FDI appears to be the best IV iron preparation compared to IS and FCM, which can lower the clinical, humanistic, and economic burden of patients with ID and IDA. FDI thus fulfills the six dimensions (efficacy, safety, economy, innovation, suitability, and accessibility) outlined in the National Health Commission’s Guidelines for the Management of Comprehensive Clinical Evaluation of Drugs and presents as a promising treatment option for ID and IDA in China.
